# The Band-Gap Modulation of Graphyne Nanoribbons by Edge Quantum Entrapment

**DOI:** 10.3390/nano8020092

**Published:** 2018-02-07

**Authors:** Yonghui Liu, Maolin Bo, Chang Qing Sun, Yongli Huang

**Affiliations:** 1 Key Laboratory of Low-Dimensional Materials and Application Technologies, Ministry of Education, Hunan Provincial Key Laboratory of Thin Film Materials and Devices, School of Materials Science and Engineering, Xiangtan University, Xiangtan 411105, China; yonghui_liu3@163.com; 2 College of Mechanical and Electrical Engineering, Yangtze Normal University, Chongqing 408100, China; bmlwd@yznu.edu.cn; 3 School of Electrical and Electronic Engineering, Nanyang Technological University, Singapore 639798, Singapore

**Keywords:** bond relaxation, band-gap, charge entrapment, edge-undercoordination, GYNRs

## Abstract

Using ab initio calculation coupled with the bond-order-length-strength (BOLS) approximation, we investigate the configurations and electronic properties of (*α*, *β*)-graphyne nanoribbons (GYNRs) with armchair (AGYNRs) and zigzag (ZGYNRs) edges. Our investigation shows that the armchair-edged *β*-GYNRs and all *α*-GYNRs are semiconductors with suitable band-gaps, and that their band-gaps increase as the widths of nanoribbons decrease; on the other hand, zigzag-edged *β*-GYNRs appear to be zero-band-gap materials. Observation results suggest that (i) atomic undercoordination shortens and stiffens the C–C bond, which contributes to the Hamiltonian and hence widens the band-gap intrinsically; (ii) zigzag-edged *β*-GYNRs lack a band-gap due to the edge-undercoordinated atoms lacking the energy to open the *β*-graphyne gap; and (iii) the edge-undercoordination of atoms occurs during charge entrapment.

## 1. Introduction

Carbon, the element most responsible for life on earth, has been proposed as a candidate material for emerging electronics [1,2]. In particular, graphene is a promising carbon-based electronic material that has attracted tremendous research interest [3,4,5,6]. The exotic electronic structure of graphene is characterized by the existence of Dirac cones, where electron and hole spectra meet linearly at single points in momentum space, called Dirac points, and charge carriers behave as massless Dirac fermions that travel at a speed of 10^6^ m·s^−1^. The existence of Dirac cones is also responsible for an anomalous quantum Hall effect and high-temperature superconductivity [7,8]. Dirac cones and their associated transport properties had until recently been considered a unique feature of graphene related to its hexagonal symmetry.

Now that graphyne has been found as a new allotrope of carbon that contains sp- and sp^2^-hybridized carbons [9,10], it has been the subject of little yet continuing interest among structural, theoretical, and synthetic scientists due to its unknown electronic, optical, and mechanical properties, as well as the proposed practical strategies for synthesizing it [11,12,13,14]. For instance, it has been found that *α*-graphyne is a semiconductor with a narrow band-gap [15,16,17]. Nevertheless, Malko et al. [18] found that *β*-graphyne and other graphyne derivatives can behave as gapless semiconductors similar to graphene, extending the application scope of graphyne in nanoelectronics and photoelectronics. Moreover, Li et al. succeeded in preparing a larger-area film of graphdiyne, which belongs to the graphyne family [19]. Much theoretical research has also been conducted on the electronic properties of graphyne and its family. This exciting experimental progress has raised hopes of synthesizing graphyne and stimulated much theoretical research on its properties, including its band-gap [20,21], charge mobility [16], and lithium-storage capacity [22,23], as well as those of related structures. Meanwhile, graphyne nanoribbons (GYNRs) can be obtained by cutting a sheet of graphyne [21,24,25,26]. The electronic and optical properties of GYNRs are quite different from those of monolayer graphyne. Thus, exploring the potential properties of GYNRs would be an interesting scientific subject.

In this presentation, we employ ab initio calculation and bond order-length-strength (BOLS) [27,28,29] simulation to describe the electronic and optical performance of (*α*, *β*)-GYNRs, armchair GYNRs (AGYNRs), and zigzag GYNRs (ZGYNRs). We also attempt to gain physical insight into bonding identities (length and energy). Instead of the conventional approach, we confirm that the extents of GYNRs’ band-gaps vary with the tunable fraction of edge-undercoordinated atoms in the edge up to three atomic layers in depth. Moreover, the analytical results also yield quantitative information about the bond length (*d_j_*), bond strength (*E_j_*), and bond-nature indicator *m*, as well as their interdependences. This quantity confirms theoretical expectations and provides guidelines for nanoscale materials and device design.

## 2. Principles

### 2.1. Size Effect and BOLS Approximation

**Figure 1:**
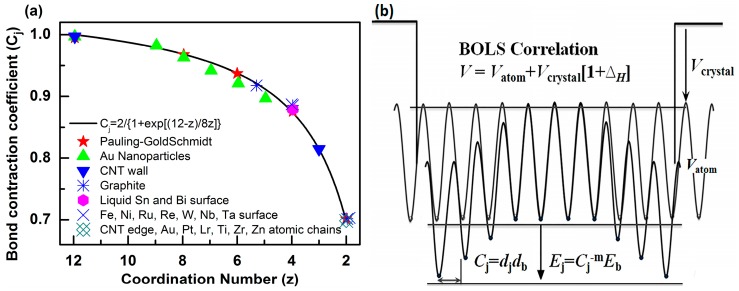
(**a**) The bond-order-length-strength (BOLS) approximation (solid line) represents the atomic “*CN*-radius” convention of Pauling [33] and Goldschmidt [34], with further evidence (scattered symbols) obtained from nanomaterials and surfaces; (**b**) Schematic illustration of broken bond-induced local strain and energy entrapment at the terminating edges, up to three atomic layers deep.

The BOLS approximation suggests that, if one bond breaks, the remaining bonds between the undercoordinated atoms become shorter and stronger. The bond length decreases from the bulk value *d_b_* to *d_j_* = *C_j_d_b_*, while the bond energy increases from the bulk value *E_b_* to *E_j_* = *C_j_*^−*m*^*E_b_*. Here, *C_i_* is the coefficient of bond contraction, *C_j_* = *d_j_*/*d_b_* = 2(1 + exp[(12 − *z*)/(8*z*)])^−1^ [30,31,32], which follows Goldschmidt’s atomic “coordination-size” convention—see Figure 1. The bond-nature indicator *m*, which correlates the bond length with the bond energy, is not freely adjustable for a given structure. Therefore, *m* is constant for different vibrational modes in one substance, while it is different in different materials. The subscripts *j* and b denote an atom in the *j*th atomic layer and in the bulk, respectively. Furthermore, *z* denotes the effective coordination number (*CN*) of the *j*th atomic layer. *j* counts inward from the outermost atomic layer up to three.

We explored the size-effect band-gap expansion of GYNRs using the BOLS approximation and confirmed that the size trend is dominated by a limited number (≤3) of edge atoms, while bonds in the core retain their bulk nature. Considering the bonds between atoms in the outermost three atomic layers, we can formulate the following relationship for the crystal potential and the associated band-gap of a nanosolid containing all edge atoms experiencing a perturbation, Δ*_R_* [35]:

(1) $$\{\begin{array}{cc} \Delta_{R} & =\frac{V_{cry}\left(N\right)-V_{cry}\left(\infty \right)}{V_{cry}\left(\infty \right)}=\frac{E_{g}\left(N\right)-E_{g}\left(\infty \right)}{E_{g}\left(\infty \right)}\\ & =\underset{j\leq 3}{\sum }\gamma_{j}\left(\frac{E_{j}}{E_{b}}-1\right)=\underset{j\leq 3}{\sum }\gamma_{j}\left(C_{j}^{-m}-1\right)\\ \gamma_{j} & =\frac{V_{j}}{V}=\tau C_{j}N^{-1} \end{array}$$

Here, *V_j_* is the volume of the *j*th layer and *N* is the dimensionless size or the atomic number of a layer measured along the radius of a sphere or across the thickness of the layered material or thin film. *γ_j_* is the surface-to-volume ratio, and *τ* is the dimensionality, given by *τ* = 1 for a nanoribbon. *E_j_* and *E_b_* are the cohesive energies per bond for an atom in the *j*th atomic layer and in the bulk at ambient pressure and 0 K.

### 2.2. Calculations and Experimental Details

In order to verify the BOLS prediction that edge-undercoordinated atoms induce size-dependence in the band-gaps of GYNRs, we obtain the band-gap energies of several GYNRs with different sizes through ab initio calculation. The calculation method used the general-gradient approximation (GGA) expressed by the Perdew-Burke-Ernzerh functional (PBE) [36], as included within the Vienna Ab initio Simulation Package (VASP) package [37,38]. In our calculation, all atoms were completely relaxed such that the energy converged at 1.0 × 10^−6^ eV/atom, and the force on each atom converged to less than 0.02 eV/Å. Optimization of the lattice vectors and atomic positions with a 1 × 9 × 1 Monkhorst-Pack *k*-mesh and an energy cutoff of 450 eV yields lattice constants of 6.92 Å and 6.97 Å for (*α*, *β*)-graphyne. A vacuum layer of 15 Å is added along the *z* direction to avoid interaction between adjacent graphyne layers.

## 3. Results and Discussion

### 3.1. Optimized Structures of (α, β)-GYNRs

**Figure 2:**
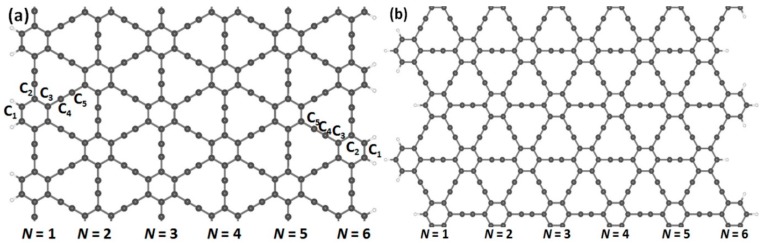

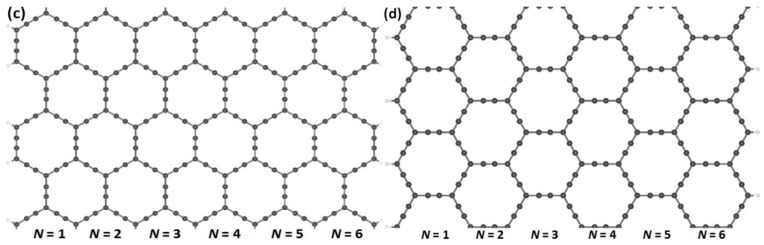
Armchair- and zigzag-edged nanoribbons can be obtained by cutting through an infinite graphyne sheet along two directions, horizontal and vertical. In (**a**,**c**), armchair-edged graphyne nanoribbons are shown with widths of *N* = 1 and 6, respectively, where *N* denotes the number of chains of carbon rings. In (**b**,**d**), zigzag-edged graphyne nanoribbons are shown with widths between *N* = 1 and 6. The gray and white spheres represent carbon and hydrogen atoms, respectively.

In order to obtain the nanoribbon structures, we optimized the two-dimensional structures of (*α*, *β*)-graphyne by choosing lattice constants of 6.92 and 6.97 Å, respectively. Cutting through an infinite graphyne sheet along two directions, indicated as horizontal and vertical, we obtained both armchair and zigzag-graphyne nanoribbons with different widths. We consider two different structures of GYNRs—see Figure 2a,c, showing armchair-edged graphyne nanoribbons, which have armchair-shaped edges, while Figure 2b,d shows zigzag-edged graphyne nanoribbons, which have zigzag-shaped edges. The dangling bonds at the edges are fully passivated by hydrogen atoms. These structures and quench-edge states often reside within the band-gap.

### 3.2. Band Structures of (α, β)-Graphyne

**Figure 3:**
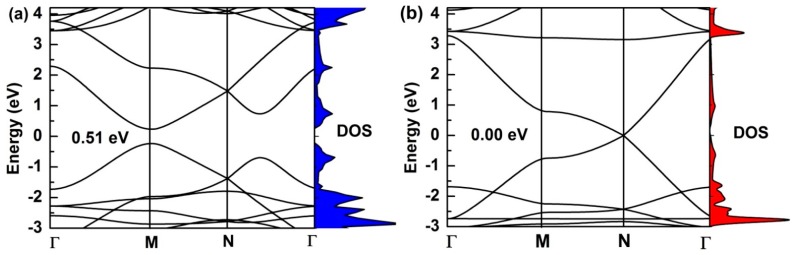
The electronic density of states and band structure for (**a**) *α*-graphyne and (**b**) *β*-graphyne. The red and blue areas represent the electronic density of states corresponding to the band structure.

The calculated electronic-band structures of monolayer (*α*, *β*)-graphyne along the high-symmetry directions of the Brillouin zone are shown in Figure 3. In the PBE-functional method, *α*-graphyne is a semiconductor with a direct band-gap of 0.51 eV (see Figure 3a), and its conduction-band minimum and valance-band maximum are located within the M point. We get very similar band structure as in the experimental results. The band-gap is found to be 0.52 eV [39,40], which is consistent with other GGA-PBE calculation. Similar calculations are performed on *β*-graphyne, as seen in Figure 3b. The valence band meets the conduction band at Dirac points, which are located at N points in the Brillouin zone. Hence, the *β*-graphyne sheet possesses different electronic properties compared to the *α*-graphyne sheet, which has a direct band-gap of 0.51 eV. Moreover, the electronic density of states of (*α*, *β*)-graphyne corresponds well to the band structure. These graphyne structures offer a good basis for the preparation of GYNRs.

### 3.3. Band Structures of (α, β)-GYNRs

**Figure 4:**
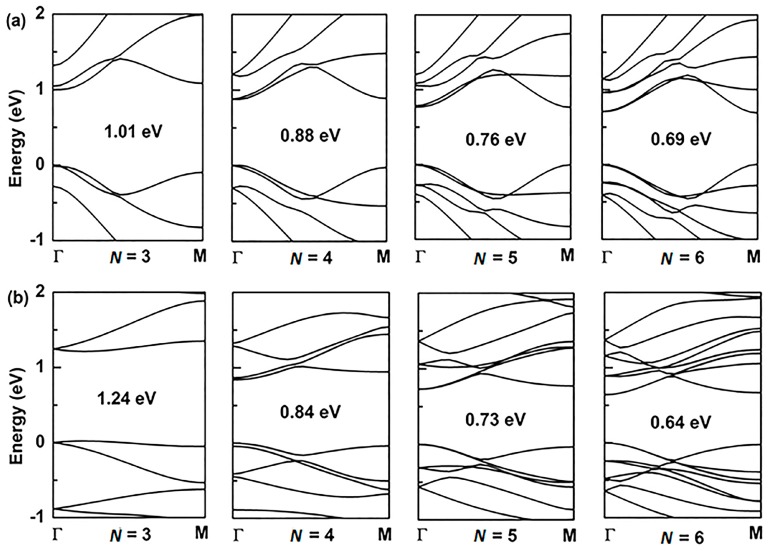
The band structure of armchair-edged *α*-graphyne nanoribbons with (**a**) *N* = 3 to 6, and zigzag-edged *α*-graphyne nanoribbons with (**b**) *N* = 3 to 6. The Fermi level is set to zero.

**Figure 5:**
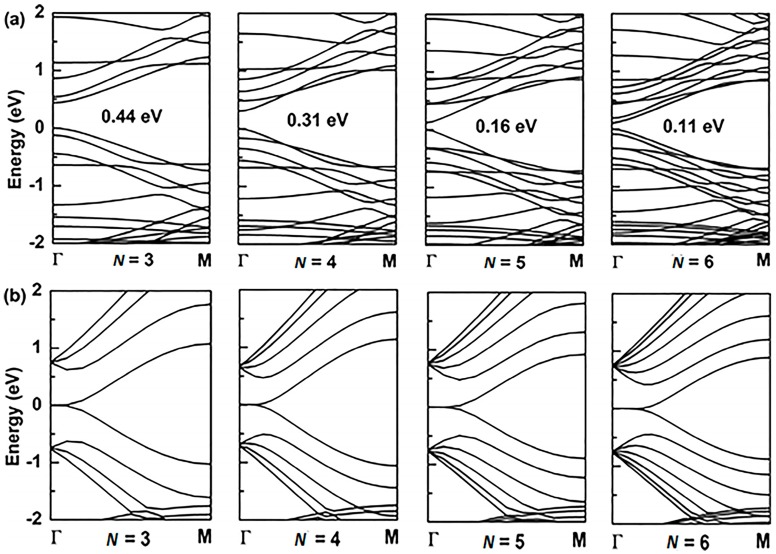
The band structure of armchair-edged *β*-graphyne nanoribbons with (**a**) *N* = 3 to 6, and zigzag-edged *β*-graphyne nanoribbons with (**b**) *N* = 3 to 5. The Fermi level is set to zero.

For the application of GYNRs to photovoltaic devices, the band-gap is a critically influential factor that directly determines the light-absorption and power-conversion efficiency. The electronic structures of the armchair- and zigzag-edged (*α*, *β*)-GYNRs are shown in Figure 4 and Figure 5, respectively, as determined by ab initio calculation. The band structures of the graphyne nanoribbons *N* = 3 to 5 (where *N* denotes the number of chains of carbon rings) were used to exhibit our calculated results. These band-gaps are markedly increased due to edge-undercoordinated atoms. In our calculations, when *N* = 3, the band-gaps of the armchair- and zigzag-edged *α*-GYNRs are respectively 1.01 and 1.24 eV (Table 1). Meanwhile, the band-gap of the armchair-edged *β*-GYNRs (*N* = 3) is 0.44 eV. However, the band-gaps of zigzag-edged *β*-GYNRs are all zero, since their edge-undercoordinated atoms lack the energy to open the gap of *β*-graphyne. These (*α*, *β*)-GYNRs all have band-gap energies larger than that of the monolayer (*α*, *β*)-graphyne with values of 0.51 and 0.00 eV, respectively, since edge-undercoordinated atoms shorten and stiffen the chemical bonds of (*α*, *β*)-GYNRs. Moreover, the carrier mobility of these graphyne nanomaterials has been calculated. The intrinsic carrier mobility of *α*-graphyne at room temperature had been confirmed to reach 5.41 × 10^5^ and 4.29 × 10^5^ cm^2^·V^−1^·s^−1^ for electrons and holes, respectively [12]. Long et al. [16] found that the electron mobility of graphdiyne can reach the order of 2.08 × 10^4^ cm^2^·V^−1^·s^−1^. They proved that the armchair-edged graphdiyne nanoribbons are more favorable than the zigzag-edged graphdiyne nanoribbons for electron transport. Graphyne and graphdiyne have similar structures since both of them are members of the graphyne family, so we assume that A*α*GYNRs is also more favorable than Z*α*GYNRs for electron transport. Finally, we conclude that the emergence of the band-gap of the zigzag-edged *β*-GYNR is an anomalous phenomenon, but comparison between the BOLS-predicted and ab initio calculated band-gaps confirm that edge-undercoordinated atoms widen the band-gap energy of (*α*, *β*)-GYNRs.

### 3.4. Edge-Atomic-Charge Entrapment

**Figure 6:**
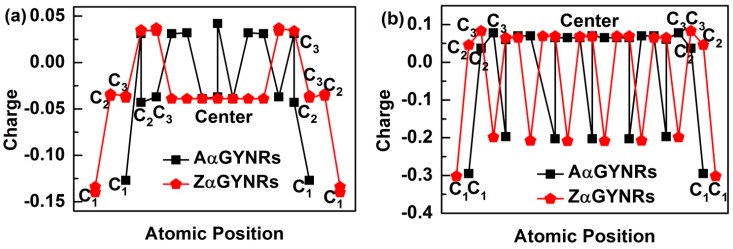
The charge entrapment of graphyne nanoribbons (GYNRs) with (**a**) armchair- and zigzag-edged *α*-GYNRs; (**b**) armchair and zigzag-edged *β*-GYNRs.

In order to understand the induced charge entrapment of edge-undercoordinated atoms of GYNRs, we have performed ab initio calculations of the geometrical structure and obtained the profiles of the Mulliken charge along the (*α*, *β*)-GYNRs nanoribbons’ width direction, as shown in Figure 6. We defined the position of carbon atoms along the GYNRs width in Figure 1a; the outermost edge of the carbon atom is *C*_1_, and so on. The Mulliken-charge distributions are symmetric on each side of the center along the width direction. The Mulliken-charge distributions at different atomic locations in GYNRs are similar to those of the broken-bond induced local strain and energy entrapment at the terminating edges up to three atomic layers—see Figure 1b. This phenomenon proves that the Mulliken-charge distribution of the edge atoms causes the entrapment phenomenon. Consistency between BOLS-predicted curves and ab initio calculated data confirms that edge-undercoordinated atoms cause charge entrapment. 

### 3.5. Size Dependence of the Band-Gap

The size-dependent band-gap of the GYNRs was calculated using the following equation:

(2) $$E_{g}=E_{g0}+\frac{E_{g0}}{N}\underset{j\leq 3}{\sum }C_{j}\left(C_{j}^{-m}-1\right)$$

**Figure 7:**
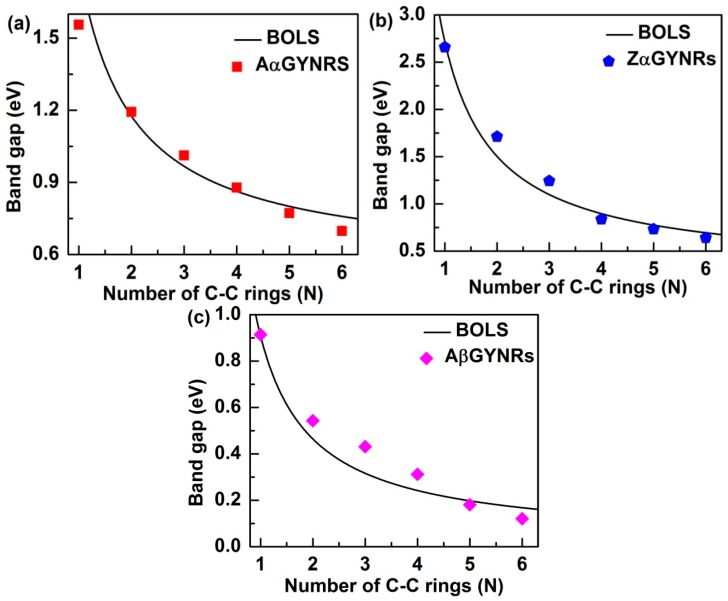
Comparison of the calculated size effect in GYNRs between ab initio and BOLS with (**a**) armchair- and (**b**) zigzag-edged *α*-GYNRs, as well as (**c**) armchair-edged *β*-GYNRs.

Equation (2) shows that the band-gap is a function of the number of chains of graphyne rings (*N*). In this equation, however, the bond-nature indicator *m* is not freely adjustable for a specific structure to correlate bond length and strength. The bond-nature indicators of (*α*, *β*)-GYNRs are calculated as *m_α_* = 5.66 and *m_β_* = 7.33. Based on these optimized *m* values, we can plot and compare the BOLS curves with the ab initio-based band-gap energies of the GYNRs—see Figure 7. The necessary parameter for the reference band-gap energy, *E_g_*_0_, was obtained. *E_g_*_0_ values for armchair- and zigzag-edged (*α*, *β*)-GYNRs were optimized to yield 0.38, 0.31, and 0.04 eV. These numbers are very close to the band-gap energy of (*α*, *β*)-graphyne. Further refinement of *m* and *E_g_*_0_ was obtained by carefully matching ab initio calculation to the entire GYNRs-width range.

A comparison between ab initio calculations and the BOLS-prediction results for the size dependence of the band-gaps of (*α*, *β*)-GYNRs is shown in Figure 7. When *α*-GYNRs and *β*-GYNRs have the same number of C–C rings, the band-gap energy of *α*-GYNRs is generally greater than that of *β*-GYNRs as seen in Figure 7a–c. There are two main reasons for this phenomenon. First, the *β*-graphyne possesses different electronic properties (0.00 eV) compared to the *α*-graphyne, which has a direct band-gap of 0.51 eV. Second, the edge-undercoordinatied atoms of *β*-GYNRs not only need to provide a certain amount of energy to open the band-gap energy of *β*-graphyne but also support the edge quantum entrapment energy, so the band-gap energy of *β*-GYNRs is less than that of *α*-GYNRs. However, ab initio-calculated band-gap energy is consistent with BOLS calculated curve. The reference band-gap energy *E*_g0_ and the bond-nature indicator *m* have been obtained and optimized. These quantities can be used to accurate match the BOLS approximation, which indicates that these size-dependent quantities originate from relaxation of the bond in the outermost atom of (*α*, *β*)-GYNRs up to a thickness of three atomic layers. The reproduction of these quantities confirms the importance of edge-undercoordinated atoms, supporting suggestions that the origin of these novel properties is merely skin thick.

## 4. Conclusions

We have developed an analytical expression that connects the ab initio calculated band-gap, the bonding identities of the samples, and the responses of the bonding identities to applied stimuli. This improves our understanding of the atomic origin of the band-gap due to edge-undercoordinated atoms in semiconductor nanocrystals. Good agreement between ab initio and BOLS confirms that, with the increase in graphyne-nanoribbon size, the edge-undercoordinated atoms widen the band-gap energy, leading to charge-entrapment in (*α*, *β*)-GYNRs. These novel discoveries demonstrate the importance of the presented approach and emphasize the role of electron spectroscopy, which permits the determination of quantitative information concerning the bonds in this and other semiconductors.

## Figures and Tables

**Table 1:** The band-gap energies for various configurations of the armchair- and zigzag-edged (*α*, *β*)-GYNRs and the monolayer (*α*, *β*)-graphyne.

Stimuli	A*α*-GYNRs	Z*α*-GYNRs	A*β*-GYNRs	Z*β*-GYNRs
*N* = 1	1.55	2.66	0.92	0.00
*N* = 2	1.29	1.72	0.54	0.00
*N* = 3	1.01	1.24	0.44	0.00
*N* = 4	0.88	0.84	0.31	0.00
*N* = 5	0.76	0.73	0.17	0.00
*N* = 6	0.69	0.64	0.11	0.00
bulk	0.51		0.00	
